# Bears habituate to the repeated exposure of a novel stimulus, unmanned aircraft systems

**DOI:** 10.1093/conphys/coy067

**Published:** 2019-01-15

**Authors:** Mark A Ditmer, Leland K Werden, Jessie C Tanner, John B Vincent, Peggy Callahan, Paul A Iaizzo, Timothy G Laske, David L Garshelis

**Affiliations:** 1 Department of Fisheries, Wildlife & Conservation Biology, University of Minnesota, 135 Skok Hall, 2003 Upper Buford Circle St. Paul, MN, USA; 2 Department of Plant and Microbial Biology, 140 Gortner Laboratory, 1479 Gortner Avenue, University of Minnesota, St. Paul, MN, USA; 3 Department of Ecology, Evolution, and Behavior, 140 Gortner Laboratory, 1479 Gortner Avenue, University of Minnesota, St. Paul, MN, USA; 4 Independent researcher, Seattle, WA, USA; 5 Wildlife Science Center, 22830 Sunrise Rd NE, Stacy, MN, USA; 6 Department of Surgery, University of Minnesota, B172 Mayo, MMC 195, 420 Delaware Street SE, Minneapolis, MN, USA; 7 Minnesota Department of Natural Resources, 1201 E Hwy 2, Grand Rapids, MN, USA

**Keywords:** Drone, cardiac biologger, habituation, stress, bears, unmanned aerial system

## Abstract

Unmanned aircraft systems (UAS; i.e. ‘drones’) provide new opportunities for data collection in ecology, wildlife biology and conservation. Yet, several studies have documented behavioral or physiological responses to close-proximity UAS flights. We experimentally tested whether American black bears (*Ursus americanus*) habituate to repeated UAS exposure and whether tolerance levels persist during an extended period without UAS flights. Using implanted cardiac biologgers, we measured heart rate (HR) of five captive bears before and after the first of five flights each day. Spikes in HR, a measure of stress, diminished across the five flights within each day and over the course of 4 weeks of twice-weekly exposure. We halted flights for 118 days, and when we resumed, HR responses were similar to that at the end of the previous trials. Our findings highlight the capacity of a large mammal to become and remain habituated to a novel anthropogenic stimulus in a relatively short time (3–4 weeks). However, such habituation to mechanical noises may reduce their wariness of other human threats. Also, whereas cardiac effects diminished, frequent UAS disturbances may have other chronic physiological effects that were not measured. We caution that the rate of habituation may differ between wild and captive animals: while the captive bears displayed large initial spikes in HR change (albeit not as large as wild bears), these animals were accustomed to regular exposure to humans and mechanical noises that may have hastened habituation to the UAS.

## Teaser

Drones are powerful new tools used in several biological sciences. Previous work indicated that animals behave fearfully or show a stress response near drone flights. Using heart monitors to gauge stress, we found that bears habituated to drones after ~20 flights over a 3–4-week period and remained habituated.

## Introduction

The human footprint continues to expand, reducing available habitat and increasing the frequency of interactions between wildlife and human activities (Venter *et al.*, 2016). Investigations of anthropogenic effects on wildlife often rely on interpreting animal behavioral responses. Recent advances in biologging technology offer the capability to sense the physiological responses of wildlife that may not be apparent from behavioral responses alone (Ditmer *et al.*, 2015).

The popularity of unmanned aircraft systems (UAS; i.e. ‘drones’) among recreationalists, researchers and conservationists has increased tremendously in recent years (Anderson and Gaston, 2013) and represents a new potential stress to wildlife (Ditmer *et al.*, 2015; Mulero-Pázmány *et al.*, 2017). As hurdles to use of UAS are eased (Vincent *et al.*, 2015; Werden *et al.*, 2015), UAS technology is seeing more use in population surveys (Seymour *et al.*, 2017; Witczuk *et al.*, 2017; Hodgson *et al.*, 2018), collection of biological samples (Wolinsky, 2017; Domínguez‐Sánchez *et al.*, 2018) and fine-scale habitat data (Olsoy Peter *et al.*, 2018), observations of behaviors (Schofield *et al.*, 2017; Barnas *et al.*, 2018b) and curbing poaching (Mulero-Pázmány *et al.*, 2015; O’Donoghue and Rutz, 2016). In the near future, UAS will be able to track animals using thermal signatures or VHF tags (Bayram *et al.*, 2016, 2017; Cliff *et al.*, 2015, 2018) and UAS swarms will be used to automatically identify multiple individuals or search larger areas for the presence of wildlife efficiently (Allan *et al.*, 2018).

However, UAS may disturb animals more than other aerial survey methods due to the very nature of what makes these devices useful: the ability to fly and hover at low altitudes. Indeed, numerous studies have observed responses of wildlife to UAS (Pomeroy *et al.*, 2015; Vas *et al.*, 2015; Brisson-Curadeau *et al.*, 2017; Mulero-Pázmány *et al.*, 2017; Barnas *et al.*, 2018a) and the noise may impact non-target species (Scobie and Hugenholtz, 2016). Work by our group (Ditmer *et al.*, 2015) documented acute stress responses in wild American black bears (*Ursus americanus*) to low-altitude UAS flights. Bears in the study often did not display behavioral signs of fear (e.g. fleeing) but in extreme cases, their heart rates nearly quadrupled (162 beats per minute) compared to pre-flight baseline data (41 beats per minute). Here, we conducted experiments to address three follow-up questions that arose from our original study: (1) do bears habituate to the presence of UAS, (2) if so, over what timescale does tolerance develop and (3) does tolerance persist in the absence of exposure to the stimulus? Chronic impacts of the use of UAS are of biological and ethical concern, especially if UAS are used in wildlife surveys designed to minimally disturb animals.

## Methods

### Field methods

We flew an Iris+ model quadcopter UAS (3D Robotics, Berkeley, CA, USA) over five adult captive American black bears (three females and two males) fenced together in a 372-m^2^ facility maintained by the Wildlife Science Center (WSC) in east-central Minnesota. The WSC is an educational, non-profit organization that provides husbandry and regular veterinary care to bears used for educational and research purposes. The study subjects had varied backgrounds prior to being housed at the WSC; three came from other captive facilities, two from the wild. The WSC hosts tours, and staff often work around the enclosure, so the bears were accustomed to both human presence and mechanical noises, such as lawnmowers.

We measured responses of bears using an implanted cardiac biologger developed for human use (Medtronic plc, Reveal XT—Generation2 with BearWare, Minnesota, USA; see Laske *et al.* 2018 for device details). We are cognizant that both the implantation of these devices and the experimental UAS flights constituted a disturbance to these bears. Wildlife research often causes some amount of disturbance or discomfort to study subjects that needs to be weighed against scientific gains (Putman, 1995; McMahon *et al.*, 2012). Our research team has successfully implanted and deployed hundreds of these cardiac biologgers in wild American black bears to gain insights about bear physiology, responses to environmental stressors, and advancements in human medicine (Laske *et al.*, 2018). The miniaturization of the cardiac biologging devices (Wilson *et al.*, 2015; Laske *et al.*, 2018), along with their tested use in humans, combined with the extraordinary healing abilities of bears (Iaizzo *et al.*, 2012), enabled the animals to quickly and fully recover from device implantation. We are consistently looking to improve our methods to reduce the potential harm to individuals. Moreover, we have tracked a number of individual wild bears with implanted cardiac biologgers over successive years and checked their health annually, finding that they showed no differences compared to non-implanted bears in terms of body condition and reproduction (Laske *et al.*, 2017, 2018). Given the rapidly expanding use of UAS as a tool to study a variety of aspects of wildlife ecology, we believed that the use of the biologgers was warranted because they enabled us to investigate physiological responses that otherwise would not have been possible, and our findings can directly help to inform best practices that could reduce animal disturbance in the long run (McMahon *et al.*, 2012).

In March 2016, bears were anesthetized as part of an animal handling course at the University of Minnesota with a mixture of Ketamine–Xylazine and a biologger was implanted subcutaneously in a peristernal location using aseptic techniques. The devices recorded each heartbeat and provided 2-min averages of heart rate (HR) in beats per minute (bpm). Data from the devices were downloaded telemetrically when the bears were anesthetized a year later for transport to a new WSC facility. Later, we matched the timing of UAS flights with date/time stamps on the HR data.

We conducted experimental UAS flights during two seasons, April–May 2016 (spring) and September–October 2016 (fall). A Federal Aviation Administration-certified pilot flew the UAS over the bears’ enclosure twice-weekly (3 or 4 days apart) for four consecutive weeks in each season, in accordance with our Certificate of Authorization (2015-CSA-150-COA). Each flight day consisted of five, 5-min flights, with 10-min pauses between flights (4 weeks each season × 2 days per week × 5 flights per day = 40 flights per season). The flight plan for all 80 flights included three distinct locations over the enclosure where the UAS hovered in place (‘loitering’) for 30 s. We designed the flight path to maximize the areal coverage of the enclosure. On each flight, the UAS completed this circuit three times at an altitude of 15 m before returning to the launch site, which was not visible to the bears. From the edge of the bear enclosure, we recorded the maximum sound pressure levels (dB SPL; re 20 μPa, Root Mean Square (RMS), A-weighted) during 4 flights using an Extech model 407 750 sound level meter. The average maximum value measured for all loitering and transition maneuvers was 60.3 dB (range 54.3–70.4 dB).

We operated our UAS at a low altitude, specifically aiming to elicit a cardiac response from which we could monitor a process of habituation (attenuated response) over time following repeated exposure. Our primary concern was not to determine the distance or flight approach that causes a physiological response. While we acknowledge that UAS operators rarely fly this close to target species, we chose to do so because we anticipated that the captive bears, living in an environment with regular human disturbances, might require a greater stimulus to achieve the same physiological reaction as wild bears in our previous study (Ditmer *et al.*, 2015), which was the baseline we were attempting to emulate. Because the HR data were not retrieved until the study was over, we could not adapt the study design to responses of the bears and hence needed to ensure that the initial flights over the bears would elicit a response.

Furthermore, we note that in selecting flight altitudes, UAS operators must balance the tradeoff between sought-after image resolution and avoiding disturbance to wildlife species, but this is complicated by the fact that disturbance distance is unknown for many species due to varying aural capacities and hearing thresholds (Scobie and Hugenholtz, 2016). Additionally, the noise of the UAS operations on any given flight can be impacted by ambient conditions and the approach path of the UAS (Vas *et al.*, 2015; Mulero-Pázmány *et al.*, 2017; Rümmler *et al.*, 2018) and species may respond physiologically but with no apparent behavioral change (Ditmer *et al.*, 2015).

Methods were approved by the University of Minnesota’s Institutional Animal Care and Use Committees (Protocol # 1002A77516) and were conducted in accordance with all relevant guidelines and regulations. Animal husbandry practices at the WSC were approved by the Institutional Animal Care and Use Committees (UMN-005).

### Statistical methods

We calculated the 95% confidence interval of HR for each bear during the 30-min period prior to the first flight of each flight day and used the upper values as the pre-disturbance baseline. We calculated differences between baselines and the maximum HRs while the UAS was in flight for each of the five flights per day (MaxHRDiff). We created two linear mixed models using R package *nlme* (Pinheiro *et al.*, 2017; R Core Team, 2018) with MaxHRDiff as the response variable in each. In Model 1, we tested whether the cardiac responses to each initial flight on a given flight day changed through time both within and between seasons. We used the first flight of each day because bears showed the strongest cardiac response to that flight. We regressed the MaxHRDiff of each first flight with Julian date and the interaction (Julian date × season) while accounting for individual differences using a random intercept based on bear identity and a random slope for Julian date. Our second model tested whether HRs changed within flight days or between the two seasons. We regressed MaxHRDiff with repetition number and the interaction of repetition number × season. We included a random intercept for bear identity. Two of the study bears were in a side enclosure during our first day of flights, so we excluded this day of data on these two bears from the analysis. We considered the bears to be habituated if the mean MaxHRDiff of the five bears was ≤10 bpm for consecutive flight days and did not subsequently increase for more than a day.

## Results

**Figure 1:**
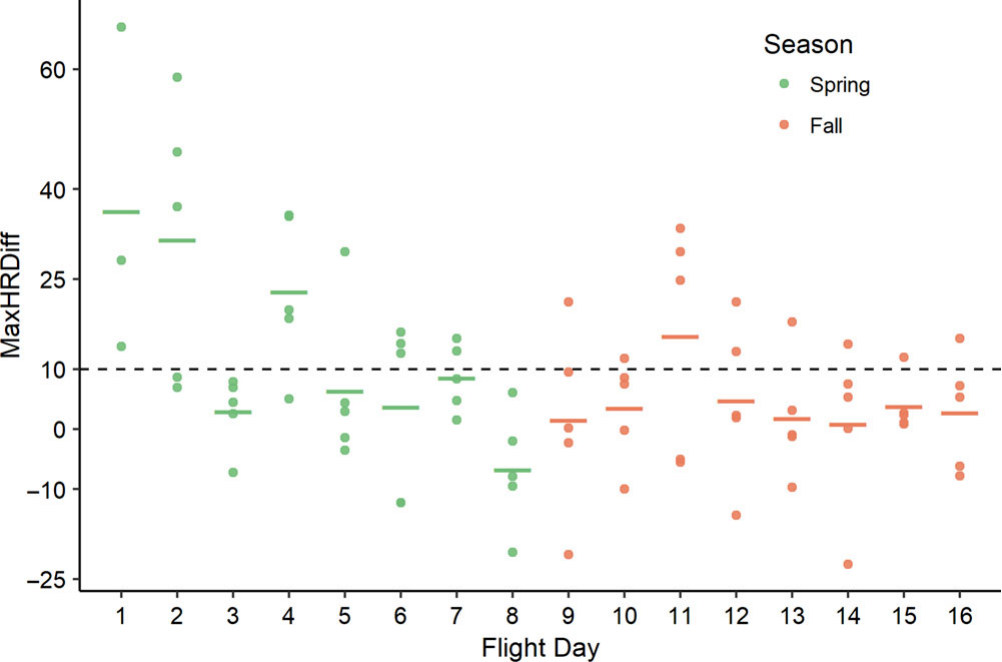
Differences between pre-flight baseline (95% upper CI of HR 30-min prior to first UAS flight of each day) and maximum HRs of five captive black bears (each point is a single bear; horizontal bars represent means of the bears) during exposure to the first UAS flight on each flight day (i.e. the flight eliciting the greatest response; see Fig. 2). We flew a quadcopter UAS 15 m over the bears’ enclosure 2 days per week, 4 weeks per season during spring and fall (16 flight-days). We considered habituation to have occurred when the mean of maximum elevations in HR remained below +10 bpm (dashed line). This occurred on flight day 5 (third week).

All bears showed at least one strong HR elevation in response to the presence of the UAS overhead (max. increase above baseline [MaxHRDiff] observed during any flight exposures: $\bar{X}$ = 52.7, range = 41−73 bpm). Responses to the initial flights each day diminished through the spring season (Fig. 1; Julian: $\hat{\beta }$ = −1.38, SE = 0.28, *P* value <0.001) and bears were considered habituated by the third week of flights (flight days 5 and 6; Fig. 1). Although it was not until the fourth week (flight days 7 and 8) when most individuals exhibited HR increases <10 bpm (Fig. 1). Anecdotal behavioral observations corroborate these findings (Supplementary Video S1). When flights resumed in the fall, HR responses to first flights of each day were similar to those at the end of the spring season (Fig. 1; season [fall]: $\hat{\beta }$ = −155.84, SE = 81.3, *P*-value 0.059;). Exposure days through the fall had much smaller effects than in spring (Julian × season [fall]: $\hat{\beta }$ = 1.25, SE = 0.38, *P* value 0.002).

**Figure 2:**
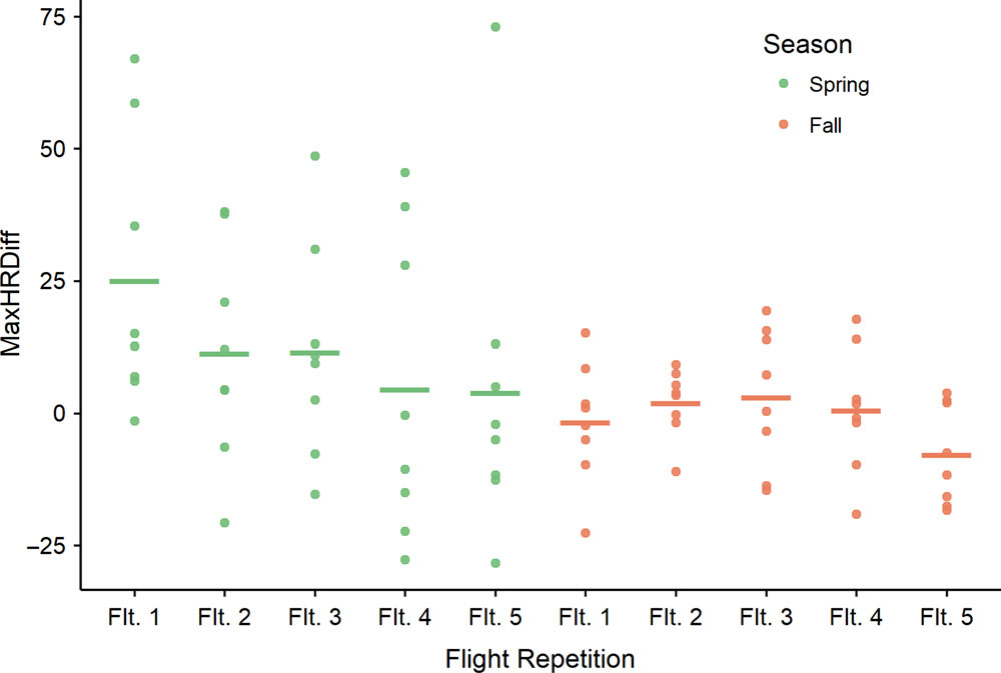
Example of diminishing cardiac responses (difference from baseline [95% upper CI of HR 30-min prior to first UAS flight of each day]) of one of five captive American black bears to repeated UAS flights. Bears were exposed to 80 flights: five times per day on eight flight days in both spring and fall. Each point is the HR response to one of these eight flights (bars represent means) grouped by the first to fifth flight of each day for each season separately.

Bear HR responses moderated from the first to fifth flight within each flight day during the spring (Fig. 2; $\hat{\beta }$ = Rep._*n* = 2.5_ = −4.40, −9.07, −10.37, −9.67, SE = 3.54, *P* value Rep._*n* = 2.5_ = 0.21, 0.011, 0.004, 0.007). Fall flights (not just first flights) elicited smaller responses than spring flights (season [fall]: $\hat{\beta }$ = −7.91, SE = 3.49, *P*-value 0.024), and the minor changes in HRs during fall flights did not differ among repetitions within days ($\hat{\beta }$ = season [fall] × Rep._*n* = 2.5_ = 3.11, 5.46, 5.82, 5.02, SE = 4.94, *P* value Rep. _2.5_ = 0.53, 0.27, 0.24, 0.31).

## Discussion

Black bears showed clear signs of increased tolerance to the UAS flights (Question 1), both short-term (within individual days comprising five flights, spanning 75 min; Fig. 2) and long-term (>20 accumulated flights over 3–4 weeks; Fig. 1; Question 2). Additionally, their tolerance to the flights was maintained after a hiatus of >3 months (Question 3), providing strong evidence of habituation to this previously foreign stressor (Bejder *et al.*, 2009).

The use of biologging technology has increased and enabled researchers to address increasingly more complex questions about the physiological responses of animals subjected to diverse anthropogenic stimuli (Wilmers *et al.*, 2015; Wilson *et al.*, 2015; Madliger *et al.*, 2018). Our experimental approach of pairing cardiac biologger technology with captive individuals enabled us to address novel questions by collecting physiological data at very fine time-scales (2-min averages) and repeatedly exposing individuals to the UAS stimulus at a regular schedule, which would not have been possible to execute with bears in the wild. The integration of physiological data into management and conservation is promising but nascent (Wilson *et al.*, 2015; McGowan *et al.*, 2017); here, we aimed to integrate these unique sources of data for improving the best practices of UAS use in wildlife conservation and research.

Given that wildlife are already repeatedly exposed to UAS for both research and conservation purposes, it is useful to know that bears could become habituated to frequent flights within a period of just a few weeks. Further UAS technological advancements will soon enable autonomous obstacle avoidance under forest canopies, regular tracking of VHF-tagged individuals (Bayram *et al.*, 2017; Cliff *et al.*, 2015, 2018) and multiple UAS working simultaneously to search out individuals (Allan *et al.*, 2018), all of which suggest increased disturbance to wildlife. Whereas our results indicated that the initial stress response attenuated, meaning the stimulus became less disturbing, this could entail other maladaptive consequences. For example, bears may key on road noise to alert them to the danger of crossing roads (Ditmer *et al.*, 2018); their waning response to other mechanical sounds may reduce their wariness and expose them to increased risks.

The rate of habituation is likely to be species dependent. Bears in general habituate to frequent contact with people, showing less fear after repeated exposure (Beckmann and Berger, 2003; Wheat and Wilmers, 2016); accordingly, bears may be predisposed to becoming tolerant of novel disturbances. Furthermore, our study subjects likely already had high tolerances for human activities, due to regular exposure to mechanical equipment, human interactions, and nearby vehicular traffic. Indeed, these captive bears initially responded less to UAS exposure than did wild bears—although the difference was not as large as might be expected given the background noises in their captive setting (max HR increase range among wild bears = 47–123 bpm [Ditmer *et al.*, 2015]; max HR increase range among captive bears = 44–78 bpm). Also, the rate of habituation in our study was likely more rapid than in the wild, where interaction with UAS may be less frequent. However, we note that the frequency of flights used in our study may not be extreme in comparison to situations where UAS are used as anti-poaching tools (Mulero-Pázmány *et al.*, 2014; O’Donoghue and Rutz, 2016), or where individuals are regularly surveyed in confined areas (e.g. haulouts [Pomeroy *et al.*, 2015], nests [Weissensteiner *et al.*, 2015], colonies [Hodgson *et al.*, 2018; Ratcliffe *et al.*, 2015] and island species [Ballaria *et al.*, 2016]). Additionally, as UAS flight times and communication among UAS improves with technological advances, UAS will commonly be used to repeatedly record behavioral observations (Allan *et al.*, 2018).

While our approach utilizing biologgers enabled us to examine fine temporal-scale cardiac changes, we echo the cautions of Bejder *et al.* (2009) against extrapolating this single metric of habituation to potential physiological responses that were not measured. Given the unknown chronic effects of continual disturbance (Wright *et al.*, 2007), we strongly recommend that UAS users follow proper ethical guidelines (Hodgson and Koh, 2016) when operating these aircraft near wildlife. We also call on researchers to increase efforts to mitigate behavioral and stress responses, for example, by using quieter, fixed-wing craft when possible and conducting data collection at a sensor’s maximum useful distance.

## Supplementary Material

Supplementary Data
